# Critical Electrocardiogram Curriculum: Setting the Standard for Flipped-Classroom EKG Instruction

**DOI:** 10.5811/westjem.2019.11.44509

**Published:** 2019-12-18

**Authors:** William P. Burns, Nicholas D. Hartman, P. Logan Weygandt, Shanna C. Jones, Holly Caretta-Weyer, Kristen Grabow Moore

**Affiliations:** * University of Wisconsin, BerbeeWalsh Department of Emergency Medicine, Madison, Wisconsin; † Wake Forest School of Medicine, Department of Emergency Medicine, Winston-Salem, North Carolina; ‡ Johns Hopkins Medicine, Department of Emergency Medicine, Baltimore, Maryland; § Oakland University William Beaumont School of Medicine, Department of Emergency Medicine, Rochester, Michigan; ¶ Stanford University, Department of Emergency Medicine, Palo Alto, California; || Emory University, Department of Emergency Medicine, Atlanta, Georgia

## Abstract

**Introduction:**

Electrocardiogram (EKG) interpretation is integral to emergency medicine (EM).^1^ In 2003 Ginde et al. found 48% of emergency medicine (EM) residency directors supported creating a national EKG curriculum.^2^ No formal national curriculum exists, and it is unknown whether residents gain sufficient skill from clinical exposure alone.

**Methods:**

The authors sought to assess the value of this EKG curriculum, which provides exposure to critical EKG patterns, a framework for EKG interpretation when the diagnosis is not obvious, and implementation guidelines and open access to any interested residency. The Foundations of Emergency Medicine (FoEM) EKG I course launched in January 2016, followed by EKG II in July 2017; they are benchmarked to post-graduate year 1 (PGY) and PGY2 level learners, respectively. Selected topics included 15 published critical EKG diagnoses and 33 selected by the authors.^5^ Cases included presenting symptoms, EKGs, and Free Open Access Medical Education (FOAM) links. Full EKG interpretations and question answers were provided.

**Results:**

Enrollment during 2017–2018 included 37 EM residencies with 663 learners in EKG I and 22 EM residencies with 438 learners in EKG II. Program leaders and learners were surveyed annually. Leaders indicated that content was appropriate for intended PGY levels. Leaders and learners indicated the curriculum improved the ability of learners to interpret EKGs while working in the emergency department (ED).

**Conclusion:**

There is an unmet need for standardization and improvement of EM resident EKG training. Leaders and learners exposed to FoEM EKG courses report improved ability of learners to interpret EKGs in the ED.

## BACKGROUND

Electrocardiogram (EKG) interpretation is integral to the practice of emergency medicine (EM).^1^ Few studies have been published regarding perceived EKG interpretation abilities of graduating residents from either EM program directors (PD) or residents. In 2003, Ginde et al. found 36% of EM residencies did not have formal EKG curricula and that 48% of EM PDs endorsed the creation of a national EKG curriculum.^2^ Limited evidence suggests that EKG interpretation ability improves over the course of EM residency training and accuracy for rarer EKG diagnoses remains poor.^3^ Further, limited evidence indicates that EM resident performance on assessments of EKG interpretation is low and EM residents have reported feeling that their EKG training is inadequate.^4^,^5^

No universally adopted or mandated EKG curriculum for EM residents currently exists and it remains unknown whether residents gain sufficient skill to transition to independent practice from clinical exposure alone. There is no standardization of EKG interpretation instruction at the undergraduate medical education level. It is likely that new EM residents enter training with highly variable EKG interpretation capabilities and a study of first month internal medicine residents found low overall performance and that nearly all felt their training was insufficient.^6^ This free and open access EKG curriculum was developed to address this gap.

## OBJECTIVES

The primary objective of this effort was to create a high quality, open-access, free EKG curriculum that would provide exposure to critical EKG patterns, a framework for EKG interpretation when the diagnosis is not obvious, and implementation guidelines with the intent to reach all interested residency programs in a standardized fashion.

The primary aims of assessment were the site leaders’ perceptions of appropriateness and satisfaction, as well as individual learner perceptions of satisfaction with the curriculum. Also assessed were learners’ satisfaction with the EKG I and EKG II courses and their perception of the effect of this curriculum on their interpretation of EKGs in the clinical environment. Finally, an attempt was made to study both leaders’ and learners’ perceptions of learners’ preparedness to interpret EKGs in the clinical environment at the start of residency.

## CURRICULAR DESIGN

This curriculum developed in response to a perception of variability in EM residents’ exposure to EKG interpretation instruction prior to the start of residency among the primary author’s postgraduate year (PGY)1 peers. Utilization of Kern’s Six Steps for Curriculum development allowed the evolution from what was initially an informal peer to peer educational intervention to a formalized curriculum that could be disseminated.^7^

Initially, a literature review was completed by the primary author and no available curriculum was identified that focused on EM resident learners. A targeted needs assessment was completed among PGY1 and PGY2 EM residents at the original institution. Specifically, they were surveyed regarding their formal EKG education prior to residency (lecture, small group, independent learning, elective), their preferences for future EKG education, and their comfort with specific aspects of EKG interpretation and diagnostic categories. The primary goals of the curriculum are to provide exposure to common and critical EKG patterns, a framework for EKG interpretation when the diagnosis is not obvious, and offer implementation guidelines and open access to any interested residency program in order to minimize the burden of work for residency leadership and faculty and to maximally engage adult learners.^8^

The foundation of the case topics are 15 critical EKG diagnoses as defined by Hartman et al.^5^ The primary author and two editors collectively identified 39 potential additional topics. Based on the relevance of each topic to EM practice 33 of the 39 topics were selected. Disagreement on inclusion was resolved by consensus of the majority. The EKG I course contains 24 cases divided into six units addressing fundamental concepts and is designed for PGY1-level learners. However, some programs are also using the curriculum for rotating medical students, PGY2 remediation, PGY1-4 EKG seminars, and/or advanced practice provider education. The EKG II course also contains 24 cases divided into six units addressing advanced content for PGY2-3 residents (Table 1). Each unit summary contains general approaches to EKG interpretation for common ED presentations such as syncope or ischemia as well as relevant example EKG images. The unit summaries support flipped classroom implementations by allowing learners to review summary content in advance of classroom sessions.^9^

Care was taken with regard to the selection of EKG images that best represent the learning objectives of the case and minimize diagnostic uncertainty. Images were selected by the consensus of the case author(s) and the two editors. Optional discussion questions and answers are also provided. An example discussion question for a case of Wolff-Parkinson-White with atrial fibrillation is provided below.

Question: “What medications are contraindicated in this situation?”

Answer: “Beta-blockers, calcium channel blockers, adenosine, and amiodarone are all incorrect choices as AV-nodal blockade can lead to preferential conduction down the accessory pathway with subsequent hemodynamic collapse, often from ventricular fibrillation.”

Free Open Access Medical Education (FOAM) content links are provided for all cases. The majority of FOAM links were sourced from the “Life in the Fast Lane” blog on the basis of its accessible explanations and broad content. When similar content is available on “Dr. Smith’s ECG Blog” site, links are also provided given the explanations provided are generally more in-depth.

**Figure 1:**
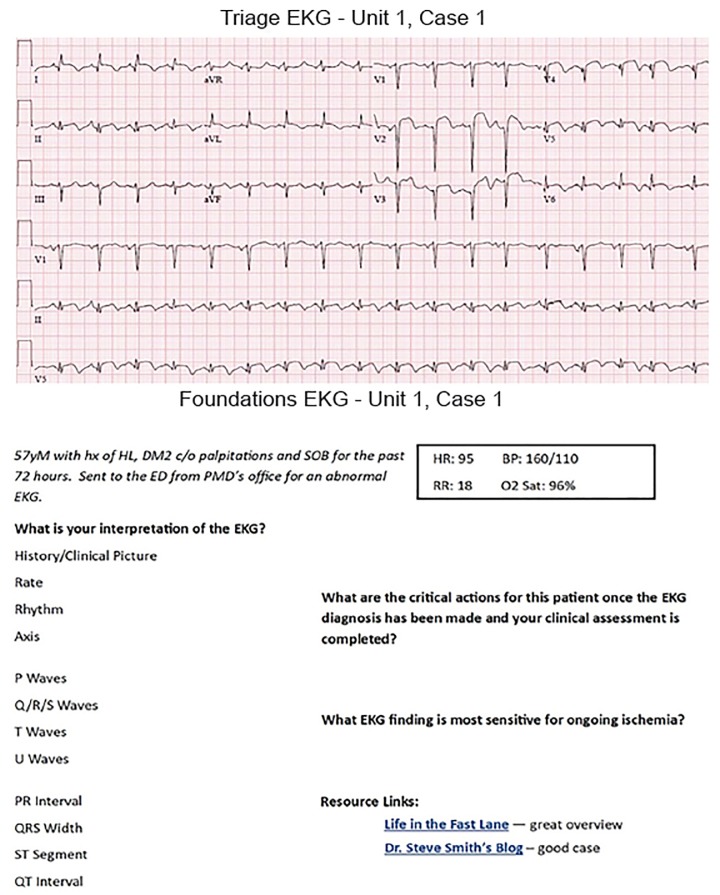
Challenge electrocardiogram, standardized interpretation stem, and questions. Challenge EKGs are provided to learners prior to Foundations meetings and contain the standardized interpretation and relevant questions. EKG, electrocardiogram.

Standardized interpretation stems and answers were modeled after the “Rule of Fours” as described by Dr. Gerard Fennessy which includes: history/clinical picture, rate, rhythm, axis, P waves, QRS morphology, T waves, U waves, PR interval, QRS width, ST segments, QT interval.^10^ Full EKG interpretations and answers to all discussion questions are provided to instructors. Figure 1 represents an example of the case layout.

Open access to all necessary course materials and best practice guidelines for implementation are provided to any interested EM residency via the Foundations of Emergency Medicine (FoEM) website www.foundationsem.com. The website contains a course schedule and links to unit summaries as well as case challenges for learners. The leader section, which provides access to EKG interpretations and answers, is password protected. Also, the provision of learner and leader specific PDFs minimizes the risk of inadvertent disclosure of answers. Leaders have the option of providing challenges either in advance of learning sessions or at the start of learning sessions. Providing the challenges in advance allows residents to attempt interpretation independently and at their own pace, while providing it during the session allows for an element of surprise that both prevents learner cooperation and mirrors the unpredictability of the clinical environment. Additionally, sites have the option of completing the review in small group settings with multiple instructors or a large group setting with a single instructor. Both courses may be implemented in either a longitudinal (15-minute review of single EKG) or workshop style (60-minute review of one unit/4 EKGs) approach.

From initial implementation in the 2014–2015 academic year the curriculum has been iteratively revised on an annual basis in response to learner and leader feedback. Such changes include the aforementioned unit summaries, FOAM links, and benchmarking of the EKG I and EKG II courses to PGY1 and PGY2 learners respectively.

## IMPACT/EFFECTIVENESS

Impact of the curriculum was primarily assessed in March 2018 via surveys of residency leaders and participating learners at sites that implemented any FoEM content. In order to continue to refine and improve the offerings by FoEM, a survey was distributed in 2018 to learners and leaders. While primarily focusing on areas for quality improvement, the impact of the curriculum on participating learners was assessed. To ensure response process validity, questions were vetted by and piloted among the FoEM leadership team. This survey was administered by the Foundations leadership; and the Emory University Institutional Review Board (IRB) deemed it IRB-exempt. The response rate for program leaders was excellent with 74 of 77 responding (96%). While learners had to self-report use of the EKG curriculum, their response rate was also acceptable at 72.2% (479/663).

Participation in the curriculum has increased rapidly alongside larger usage of Foundations of Emergency Medicine content nationally. Based on programs that registered for Foundations and completed the end-of-year survey, the number of residency programs participating in Foundations EKG increased from one program in 2014–2015 to 49 in 2018–2019 and participating resident learners increased from 15 in 2014–2015 to 1,311 in 2018–2019. Correspondingly, overall Foundations of Emergency Medicine participation increased from 1 program in 2014–2015 to 100 in 2018–2019. The last reported ACGME data regarding total EM residency programs and total EM residents was in 2018–2019 with 247 programs and 7,940 residents.^11^ During the 2018–2019 academic year, 19.8% of EM residency programs and 16.5% of EM residents were exposed to Foundations EKG as compared to 40.5% of programs that participated in any Foundations of Emergency Medicine content. Fewer than 100% of programs responded to our survey and thus these numbers may somewhat underrepresent our total impact.

Leaders and learners were surveyed regarding their satisfaction with both EKG I and EKG II (Table 2). Survey questions were designed around a five-point Likert scale (Strongly Disagree, Disagree, Neutral, Agree, Strongly Agree). 100% of responding leaders agreed or strongly agreed that they were satisfied with both EKG I (n = 37) and EKG II (n = 22). Most learners agreed or strongly agreed that they were satisfied with both EKG I (86.7%, n = 309) and EKG II (80.3%, n = 152).

The survey also assessed appropriate benchmarking of EKG I to PGY1 learners and EKG II to PGY2 learners. Leaders were asked to rate their agreement that each course was appropriate for PGY1 and PGY2 residents respectively. They reported agreeing or strongly agreeing that both were learner-level appropriate at a rate of 97.3% (n = 37) for EKG I, and 100% (n = 22) for EKG II. Learners were also surveyed regarding the perceived effect of each curriculum. We found 85.4% (n = 309) of EKG I learners and 83.2% (n = 152) of EKG II learners agreed or strongly agreed that the respective courses had “improved my ability to interpret EKGs in the clinical environment.”

An attempt was made to assess the perceptions of all FoEM leaders and learners with regard to learner preparedness to interpret EKGs in the clinical environment at the start of residency. Only 27.6% of the 1,252 learners who responded indicated that they agreed or strongly agreed with the statement: “at the beginning of residency, I was prepared to interpret EKGs.” Only 13.5% of the 73 leaders who responded indicated that they agreed or strongly agreed with the statement: “at the beginning of residency, compared to their classmates, interns are equally prepared to interpret EKGs.”

These preliminary data suggest that an unmet need for standardization and improvement of EKG training exists. Users of the FoEM EKG I and EKG II curricula report significant satisfaction and perceived benefits to patient care.

## LIMITATIONS

This work contains important limitations to consider. First, with regard to implementation, there was no specific mandate on how the curriculum had to be provided or any method for assuring equivalent quality of in-person instruction. For example, variability between sites existed between the use of a flipped classroom approach or how many cases were covered per week. All data was gathered from surveys which carries inherent potential for bias including self-reporting, response process validity, and unintentionally leading questions. It is also possible that respondents are biased in their evaluation of this curriculum based on their preceding experience with other EKG curricula given the variance in pre-existing EKG curricula at participating sites. Unfortunately, despite instructions to the have a single leader from each participating site complete the survey, 25% of sites ultimately submitted more than one survey. The issue of multiple surveys from one program was dealt with in the following manner: since the survey was anonymous, it was not possible to simply exclude extraneous surveys. For a given program, if discrepancies occurred between the multiple surveys, subjective responses were averaged to produce a composite result. For discrepancies in objective responses, attempts were made to assess which response was most valid in order to adjudicate. In assessing validity, complete responses and lower total participant counts were favored to minimize artificial inflation of impact.

## CONCLUSION

This free, open-access, standardized, flipped-classroom, critical EKG interpretation curriculum continues to be refined. This curriculum consists of two courses, EKG I and EKG II, and is designed to target appropriate PGY-level learners, minimize the work required by instructors and residency leadership, and provide a standardized curriculum to learners. Learners who have used the curriculum report high satisfaction and improvement in their perception of their individual ability to interpret EKGs in the clinical environment. Both leaders and learners alike believe that residents start residency with disparate abilities to interpret EKGs. Marked growth has occurred in the number of learners impacted by the curriculum and with continued growth it may set the standard for EM resident EKG education.

## Figures and Tables

**Table 1:** EKG I and EKG II Courses.

EKG I Course		EKG II Course	
Unit I	Approach to Ischemia: STEMI	Unit VII	Approach to Fasicular Blocks
Case 1	Anterolateral STEMI	Case 25	Bundle Branch Blocks
Case 2	Inferior STEMI	Case 26	Left Anterior Fascicular Block
Case 3	Posterior STEMI	Case 27	Left Posterior Fascicular Block
Case 4	Left Bundle Branch Block STEMI	Case 28	Bi/Trifascicular Blocks
Unit II	Approach to Ischemia: Mimics	Unit VIII	Approach to Complex Ischemia
Case 5	Benign Early Repolarization	Case 29	Diffuse STD with aVR elevation
Case 6	Left Ventricular Aneurysm	Case 30	High Lateral STEMI
Case 7	Hyperkalemia	Case 31	DeWinter ST/T complex
Case 8	Pericarditis	Case 32	Right Ventricular Infarct
Unit III	Approach to Syncope	Unit IX	Miscellaneous Ischemic EKGs
Case 9	Brugada	Case 33	Pulmonary Embolism
Case 10	Long QT	Case 34	Cerebral T-waves
Case 11	Wolff-Parkinson-White	Case 35	Wellen’s Waves
Case 12	Hypertrophic Obstructive Cardiomyopathy	Case 36	New Right Bundle Branch and Left Anterior Fascicular Blocks
Unit IV	Approach to Bradyarrhythmias	Unit X	Potassium Derangement
Case 13	2nd Degree AV Block Type I	Case 37	Hypokalemia
Case 14	2nd Degree AV Block Type II	Case 38	Mild/Moderate Hyperkalemia
Case 15	3rd Degree AV Block	Case 39	Severe Hyperkalemia
Case 16	Ventricular Escape Rhythm	Case 40	Wide Complex Bradycardia
Unit V	Approach to Tachyarrhythmias: Narrow Complex	Unit XI	Miscellaneous EKGs
Case 17	Supraventricular Tachycardia	Case 41	Antidromic Atrioventricular Reentrant Tachycardia
Case 18	Atrial Fibrillation with Rapid Ventricular Response	Case 42	Arrhythmogenic Right Ventricular Cardiomyopathy
Case 19	Atrial Flutter with Rapid Ventricular Response	Case 43	Digoxin Toxicity EKG Findings
Case 20	Multifocal Atrial Tachycardia	Case 44	Accelerated Idioventricular Rhythm
Unit VI	Approach to Tachyarrhythmias: Wide Complex	Unit XII	Approach to Paced Rhythms
Case 21	Ventricular Tachycardia	Case 45	Normal Atrioventricular Paced
Case 22	Wolff-Parkinson-White	Case 46	Normal Ventricular Paced
Case 23	Hyperkalemia	Case 47	Pacemaker-mediated Tachycardia
Case 24	Sodium Channel Blockade	Case 48	Failure to Capture

Foundations EKG I and II courses contain 48 unique cases including life threats and mimics.

*STEMI*, ST-elevation myocardial infarction; *AV*, atrioventricular; *EKG*, electrocardiogram.

**Table 2:** 2017–2018 Foundations EKG Learner and Leader Survey Results.

2017–2018 Learners Survey Results	Agree or strongly agree	Mean	N
Please respond to the following statement: At the beginning of residency, I was prepared to interpret EKGs.	27.6%	2.51	1,252
I am satisfied with the EKG I course.	86.7%	4.34	309
The EKG I course has improved my ability to interpret EKGs in the clinical environment.	85.4%	4.27	309
I am satisfied with the EKG II course.	80.3%	4.01	152
The EKG II course has improved my ability to interpret EKGs in the clinical environment.	83.2%	4.11	152

2017–2018 Leaders Survey Results	Agree or strongly agree	Mean	N

Please respond to the following statement: At the beginning of residency, compared to their classmates, interns are equally prepared to interpret EKGs.	13.5%	2.52	73
I am satisfied with EKG I course content.	100%	4.59	37
EKG I course content is appropriate for PGY-1 residents.	97.3%	4.62	37
I am satisfied with the EKG II course.	100%	4.58	22
EKG II course content is appropriate for PGY-2 residents.	100%	4.58	22
